# A Metabolic Profiling Strategy for the Dissection of Plant Defense against Fungal Pathogens

**DOI:** 10.1371/journal.pone.0111930

**Published:** 2014-11-04

**Authors:** Konstantinos A. Aliferis, Denis Faubert, Suha Jabaji

**Affiliations:** 1 Department of Plant Science, Macdonald Campus of McGill University, Sainte-Anne-de-Bellevue, Quebec, Canada; 2 Institut de Recherches Cliniques de Montréal, Montréal, Quebec, Canada; Korea University, Republic of Korea

## Abstract

Here we present a metabolic profiling strategy employing direct infusion Orbitrap mass spectrometry (MS) and gas chromatography-mass spectrometry (GC/MS) for the monitoring of soybean's (*Glycine max* L.) global metabolism regulation in response to *Rhizoctonia solani* infection in a time-course. Key elements in the approach are the construction of a comprehensive metabolite library for soybean, which accelerates the steps of metabolite identification and biological interpretation of results, and bioinformatics tools for the visualization and analysis of its metabolome. The study of metabolic networks revealed that infection results in the mobilization of carbohydrates, disturbance of the amino acid pool, and activation of isoflavonoid, *α*-linolenate, and phenylpropanoid biosynthetic pathways of the plant. Components of these pathways include phytoalexins, coumarins, flavonoids, signaling molecules, and hormones, many of which exhibit antioxidant properties and bioactivity helping the plant to counterattack the pathogen's invasion. Unraveling the biochemical mechanism operating during soybean-*Rhizoctonia* interaction, in addition to its significance towards the understanding of the plant's metabolism regulation under biotic stress, provides valuable insights with potential for applications in biotechnology, crop breeding, and agrochemical and food industries.

## Introduction

Metabolomics is a robust bioanalytical tool for the comprehensive analysis and monitoring of plant metabolome [1]–[3]. However, its application for monitoring the regulation of the global plant metabolism in response to biotic stresses is still in its infancy, receiving increasing attention [4]–[6]. This, could provide valuable information for applications in plant biotechnology, biomarker-assisted selection, and agrochemical, food, and pharmaceutical industries [7], and in turn could boost agricultural production. The recent advances in bioanalytical protocols, analyzers, metabolite databases, and bioinformatics software enable the recording of a vast number of chemical features in the analyzed plant samples, whose identification and biological interpretation is challenging. Moreover, there is an increasing demand for standardization of data reporting for large-scale metabolomics [8], which will help researchers to cross-reference results from different studies with profound benefits. Within this context, we have undertaken the task of developing a high-throughput metabolomics/bioinformatics protocol for the robust dissection of plant-fungal pathogen interaction using the pathosystem; soybean [*Glycine max* (L.) Merrill, Leguminosae] and its soil-borne fungal pathogen-*Rhizoctonia solani* Kühn (anastomosis group 4, AG4). For the analysis of soybean's metabolome direct infusion Orbitrap mass spectrometry (DIMS) and gas chromatography-MS (GC/MS) analyzers were employed, which exhibit complimentary capabilities for metabolite detection and identification.

Soybean is a crop grown on almost 6% of arable land [9] and among the most important plant sources of human food, animal feed protein, and cooking oil [10], phytoestrogens [11], and biodiesel [12]. It is the first legume species with a complete sequence [13], and therefore, a key reference for the development of high-throughput plant metabolomics protocols. Various biotic constraints such as, bacteria, fungi, nematodes, and insects threaten its production by directly reducing seed yield and/or quality [14]. Among them is the soil-borne fungal pathogen *R. solani*, which causes Rhizoctonia rot of young seedlings and is characterized by post-emergence symptoms of brown necrotic lesions observed on roots, hypocotyls and stems. In North America, severe yield losses of up to 48% result from stand reduction in newly planted fields and premature death of diseased plants that produce undersize seeds [15]. Few management options exist since no resistant or tolerant varieties are available.

During plant-pathogen interaction a sequence of chemical, molecular, and mechanical events occur, implicated in a distinctive “dialogue” leading to the development of the disease [16]. Apart from recent “omics” studies on the responses of soybean to stresses [17], [18], no study exists on the regulation of the global soybean metabolome in response to pathogen infection. In the present study, we explore whole-cellular processes at the metabolome level under a pathogen attack and present a comprehensive overview of soybean's primary and secondary metabolism regulation. This is accomplished by combining multidimensional metabolomics data in an effort to detect specific pathways indispensable for the regulation of metabolism. Key components in the approach are the construction of a standardized soybean library and the use of bioinformatics software for the visualization and analyses of soybean metabolome, which can significantly accelerate the steps of metabolite identification and biological interpretation of results.

## Results and Discussion

### Monitoring Soybean's Metabolome and its Temporal Perturbation in Response to *Rhizoctonia* Infection

**Figure 1:**
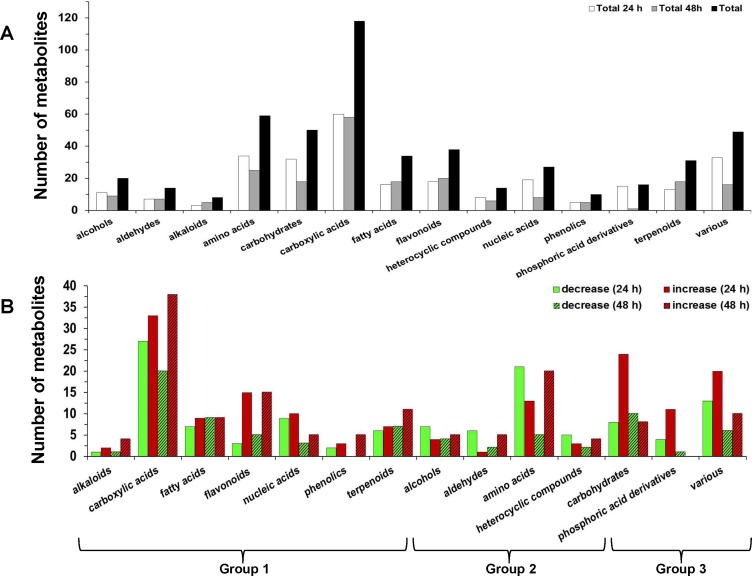
Classification of soybean metabolites into chemical groups in response to *Rhizoctonia* infection. The total number of signatory metabolites at 24 h and 48 h post-inoculation (*A*), and the total number of signatory metabolites classified into increased and decreased in infected seedlings compared to controls at 24 h and 48 h post-inoculation (*B*), are displayed. For the chemical classification of metabolites information was retrieved from the database PubChem.

**Figure 2:**
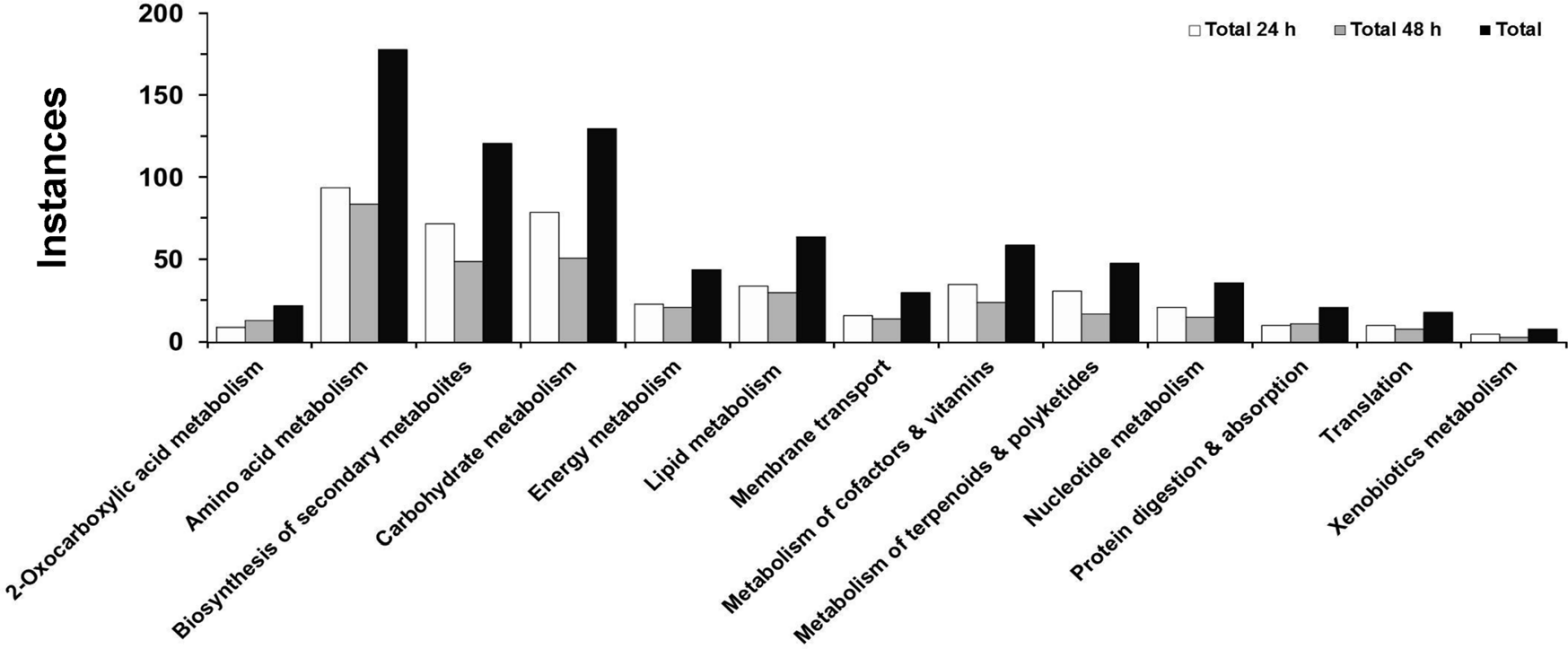
Classification of metabolites signatory of the soybean's response to *Rhizoctonia solani* invasion based on their participation in plant metabolic pathways/functions, measured as instances, since a metabolite can be involved in more than one pathway. For standardization purposes the KEGG coding system for soybean pathways is adapted. The total number of instances at 24 h and 48 h post-inoculation (*A*), and the total number of instances classified into increased and decreased in infected seedlings at 24 h and 48 h post-inoculation (*B*), are displayed.

The complexity of plants' metabolome makes their deconvolution challenging, requiring often the utilization of more than one analyzer. For DIMS-based metabolomics, ion suppression can impact the validity of analysis, however, information on its dynamics is yet fragmented. Here, analysis of samples with similar metabolite profiles resulted in consistent ion suppression as revealed by the tight clustering among biological replications performing multivariate analyses (Fig. S1). The latter confirms the potential of DIMS for high-throughput metabolomics applications in line with recent studies [19], [20]. The developed protocol (Fig. S2) enabled the in-depth deconvolution of DIMS data, as confirmed by the large number of obtained frames using the software SIEVE (Table S1). On the other hand, GC/MS analyses facilitated the construction of a matrix composed of 135 features, reproducibly detected across treatments. In total, 377 putatively or absolutely identified metabolites were statistically significant different between controls and infected soybean sprouts (Data Set S1). MS spectra of identified metabolites of biological origin from GC/MS analysis, and MS/MS spectra from DIMS Orbitrap analysis provided in the Data Set S2 and Data Sets S3 and S4, respectively. Sets of original GC/MS and DIMS Orbitrap data can be found at the public repository of Metabolights (http://www.ebi.ac.uk/metabolights/) (Accession # MTBLS118 and MTBLS117, respectively). The complexity of undergoing biochemical events during soybean-*Rhizoctonia* interaction (Fig. S3) is indicated by the diversity of chemical groups and biosynthetic pathways involved (Figs. 1 and 2, Fig. S4). Up-regulated metabolites also detected in fungal profiles, which could have leverage on data interpretation, were omitted from analyses.

Principal component analysis (PCA) was performed initially for the whole dataset revealing no outliers (data not shown). In a second step, PLS-DA revealed a strong discrimination between metabolite profiles of control and *Rhizoctonia*-inoculated seedlings (Fig. S1), reflecting their response as early as 24 h post-inoculation. Furthermore, the tight clustering among biological replications confirms the robustness and reproducibility of the experimental protocol.

### Metabolomics Data Reporting and Biological Interpretation for the Global Overview and Biological Interpretation of Fluctuations of Soybean Metabolome

Here, a protocol was developed for the robust data reporting for metabolic profiling experiments, which provides an overview of metabolome regulation (Fig. S2). Central to this approach is the construction of a soybean-specific in-house built library, in which entries for metabolites and pathways follow the coding of freely available repositories. The results reveal its applicability for robust interpretation of large metabolomics data sets (Figs. 1, 2, Table 1). Also, the use of standardized chemical classification allowed the robust synopsis of the soybean's response to pathogen attack based on the chemical groups of metabolites (Fig. 1).

**Table 1:** Classification of signatory metabolites of soybean response to *Rhizoctonia solani* invasion at 24 h and 48 h post-inoculation based on their participation in biosynthetic pathways, measured as instances, since a metabolite can be involved in more than one pathway.

Biosynthetic pathway	Decrease (24 h)	Increase (24 h)	Decrease (48 h)	Increase (48 h)	Function
map01210 2-Oxocarboxylic acid metabolism	6	3	1	12	2-Oxocarboxylic acid metabolism
map00280 Valine leucine & isoleucine degradation	1	4	0	4	Amino acid metabolism
map00290 Valine leucine & isoleucine biosynthesis	3	4	1	6	Amino acid metabolism
map00300 Lysine biosynthesis	2	1	2	5	Amino acid metabolism
map00310 Lysine degradation	0	1	0	4	Amino acid metabolism
map00330 Arginine & proline metabolism	1	4	6	3	Amino acid metabolism
map00360 Phenylalanine metabolism	6	0	1	4	Amino acid metabolism
map00380 Tryptophan metabolism	2	1	1	4	Amino acid metabolism
map00400 Phenylalanine tyrosine & tryptophan	6	3	3	5	Amino acid metabolism
map00410 b-Alanine metabolism	3	3	4	1	Amino acid metabolism
map00940 Phenylpropanoid biosynthesis	8	8	0	5	Biosynthesis of secondary metabolites
map00941 Flavonoid biosynthesis	1	5	0	1	Biosynthesis of secondary metabolites
map00943 Isoflavonoid biosynthesis	2	8	4	11	Biosynthesis of secondary metabolites
map00945 Stilbenoid diarylheptanoid & gingerol	1	3	0	1	Biosynthesis of secondary metabolites
map00960 Tropane piperidine & pyridine alkaloid biosynthesis	3	0	1	5	Biosynthesis of secondary metabolites
map00966 Glucosinolate biosynthesis	5	4	0	10	Biosynthesis of secondary metabolites
map00010 Glycolysis-Gluconeogenesis	1	5	1	1	Carbohydrate metabolism
map00030 Pentose phosphate pathway	1	5	0	3	Carbohydrate metabolism
map00040 Pentose & glucuronate interconversions	1	4	1	1	Carbohydrate metabolism
map00052 Galactose metabolism	3	4	0	3	Carbohydrate metabolism
map00053 Ascorbate & aldarate metabolism	2	5	3	3	Carbohydrate metabolism
map00500 Starch & sucrose metabolism	2	4	1	4	Carbohydrate metabolism
map00520 Amino sugar & nucleotide sugar metabolism	3	4	0	3	Carbohydrate metabolism
map04973 Carbohydrate digestion & absorption	1	3	0	3	Carbohydrate metabolism
map00073 Cutin suberine & wax biosynthesis	4	0	5	1	Lipid metabolism
map00592 *α*-Linolenic acid metabolism	0	4	0	7	Lipid metabolism
map00561 Glycerolipid metabolism	0	3	1	1	Lipid metabolism
map00564 Glycerophospholipid metabolism	1	5	1	0	Lipid metabolism
map00561 Glycerolipid metabolism	0	3	1	1	Lipid metabolism
map02010 ABC transporters	6	10	4	10	Membrane transport
map00750 Vitamin B6 metabolism	3	6	2	1	Metabolism of cofactors & vitamins
map00770 Pantothenate & CoA biosynthesis	1	5	2	3	Metabolism of cofactors & vitamins
map00780 Biotin metabolism	1	0	0	3	Metabolism of cofactors & vitamins
map04977 Vitamin digestion & absorption	1	4	2	1	Metabolism of cofactors & vitamins
map00908 Zeatin biosynthesis	2	7	1	0	Metabolism of terpenoids & polyketides
map00230 Purine metabolism	6	6	4	2	Nucleotide metabolism
map00240 Pyrimidine metabolism	5	5	5	4	Nucleotide metabolism
map04974 Protein digestion & absorption	6	4	2	9	Protein digestion & absorption
map00970 Aminoacyl-tRNA biosynthesis	7	3	2	6	Translation

For standardization purposes the KEGG classification system for soybean pathways was adapted. Pathways with the highest number of identified **signatory metabolites** are displayed.

The vast majority of identified signatory metabolites of the infection belong to carboxylic and amino acids, carbohydrates, and flavonoids (Fig. 1*A*). Clear patterns of fluctuation in the number of increased and decreased metabolites are observed at both time points (Fig. 1*B*). Based on this pattern, the chemical classes of the identified signatory metabolites are categorized into three groups: i) group 1, for which the number of metabolites that increased following inoculation was substantially higher at 24 h, and higher (or equal) at 48 h post-inoculation, compared to those that were decreased, ii) group 2, for which their number of metabolites that increased following inoculation was substantially lower at 24 h and substantially higher (or equal) at 48 h post-inoculation, compared to those that were decreased, and ii) group 3, for which their number of metabolites that increased following inoculation was higher at 24 h and lower at 48 h post-inoculation, compared to those that were decreased.

Since a given metabolite could be component of different pathways and influence multiple biological processes, the monitoring of plant's metabolic networks becomes challenging. To overcome such challenge and simplify the process, the standardized overview of pathways was achieved by adapting the KEGG classification for soybean pathways (Fig. 2 and Table 1). A general disturbance of the plant's metabolism was observed in response to infection, with the largest number of identified signatory metabolites involved in amino acid and carbohydrate metabolism, and biosynthesis of secondary metabolites. In the vast majority of instances, metabolic pathways of infected seedlings are much more active compared to those in controls (Table 1). Such observation indicates that the plant responds at early stages of infection and invokes a rapid metabolic response involving primary and secondary biosynthetic pathways.

### Visualization of the Soybean Metabolome, Metabolic Sub-Networks, and their Perturbation using Bioinformatics Software

**Figure 3:**
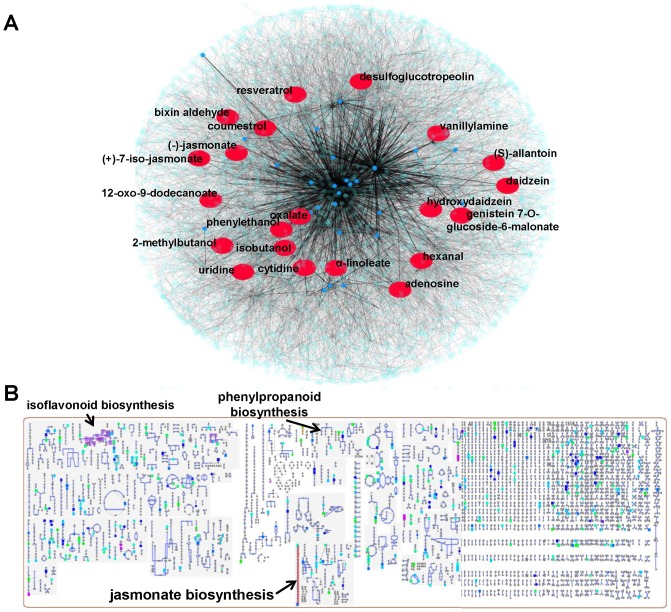
Global soybean metabolome and its perturbation in response to *Rhizoctonia solani* at 48h post-inoculation (*A*) and sub-network generated by connecting possible paths between the displayed signatory metabolites (*B*) using the software Cytoscape and the cellular overview tool of SoyCyc (*C*). Representative metabolites are indicated in red color (*A*) and (*B*), whereas in the panel (*C*), using the Data Set S2, metabolites with KEGG identifiers are highlighted.

**Figure 4:**
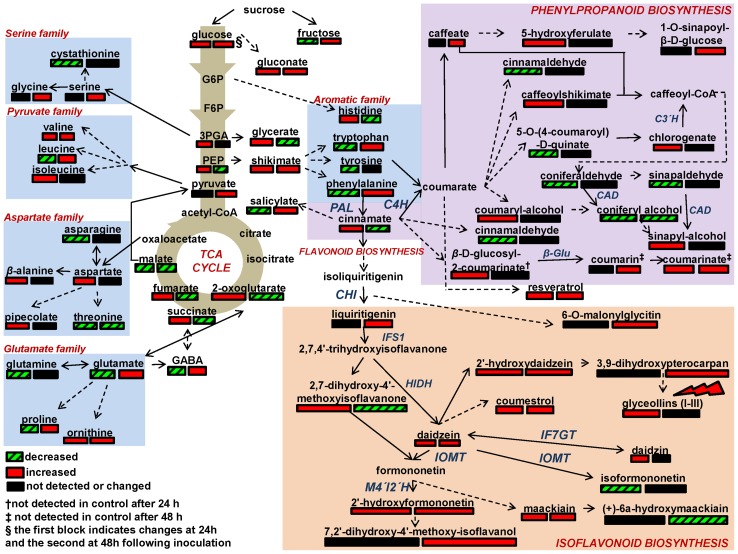
Color-coded fluctuation of the soybean's seedling metabolic network caused by *Rhizoctonia solani* infection at 24 h and 48 h post-inoculation, including portions of the amino acid biosynthesis, and the isoflavonoid and phenylpropanoid biosynthetic pathways, which were detected as important components of its defense mechanism. Genes in the reference pathway of KEGG for soybean that are hyperlinked to gene entries are displayed. Metabolite fluctuations are coded using a color code based on *P*-values (*P*<0.05) performing the Student's t-test and means of scaled and centered PLS regression coefficients (CoeffCS) from five biological replications and a quality control sample per treatment. Dashed lines symbolize multi-step or not fully elucidated reactions and solid lines one-step reactions. [3PGA; 3-phosphoglycerate, isoflavone synthase 1; C3′H; coumaroylquinate (coumaroylshikimate) 3′-monooxygenase, EC: 1.14.13.36, NCBI-GeneID: 100811080, C4H; coumarate 4-hydroxylase, EC: 1.14.13.11, NCBI-GeneID: 100499623, CAD; cinnamyl-alcohol dehydrogenase, EC: 1.1.1.195, NCBI-GeneID: 100777558, CHI; chalconeisomerase (5 isozymes), F6P; fructose-6-phosphate, G6P; glucose-6-phosphate, β-Glu; glucosidase β-glucosidase, EC: 3.2.1.21, NCBI-GeneID: 100779642, HIDH; 2-hydroxyisoflavanone dehydratase, EC: 4.2.1.105, NCBI-GeneID: 547489, IF7GT; isoflavone 7′-O-glucosyltransferase, EC: 2.4.1.170, NCBI-GeneID: 100101902, IFS1; isoflavone synthase 1, EC: 5.4.99.-, NCBI-GeneID: 100037450, IOMT; isoflavone 7′-O-Methyltransferase (14 isozymes), PAL; phenylalanine ammonia-lyase, EC: 4.3.1.24, NCBI-Gene ID: 100787902, PEP; phosphoenolpyruvate].

**Figure 5:**
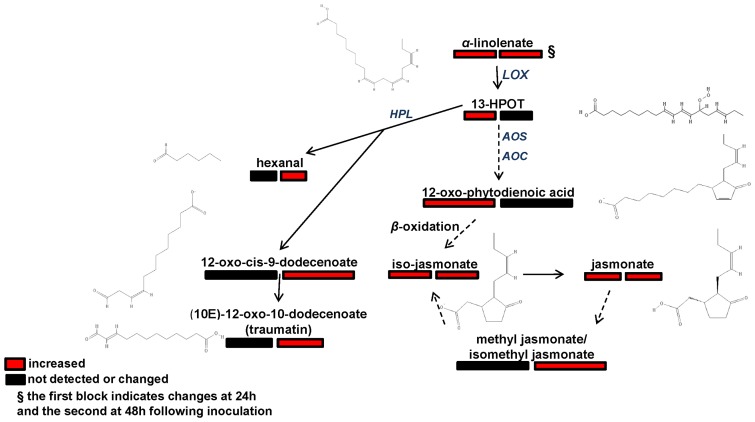
Fluctuations in the *α*-linolenate metabolism of soybean in response to *Rhizoctonia solani* at 24 h and 48 h post-inoculation coded using a color code based on *P*-values (*P*<0.05) performing the Student's t-test or means of scaled and centered PLS regression coefficients (CoeffCS) from five biological replications and a quality control sample per treatment. Genes in the reference pathway of KEGG for soybean that are hyperlinked to gene entries are displayed. Dashed lines symbolize multi-step or not fully elucidated reactions and solid lines one-step reactions [13-HPOT; (9Z,11E,15Z)-(13S)-hydroperoxyoctadeca-9,11,15-trienoate, AOC; allene oxide cyclase, EC: 5.3.99.6, NCBI-GeneID:100800036, AOS; hydroperoxidedehydratase, EC: 4.2.1.92, NCBI-GeneID: 100037481, HPL; hydroperoxidelyase, EC: 4.1.2.-, NCBI-GeneID: 100784395, LOX; lipoxygenase, EC: 1.13.11.12, NCBI-GeneID: 100785480].

The use of Cytoscape and the cellular overview tool of SoyCyc made possible the visualization of the global metabolome of soybean and *in-silico* dissection of its sub-networks. Using Cytoscape's plug-in BisoGenet [21], selected metabolites such as phytoalexins and flavonoids, and biosynthetic precursors, whose relative concentrations significantly increased at 48 h post-inoculation and possible interconnecting paths between them are highlighted (Fig. 3*A*). Also, metabolites (Data Set 4), whose relative concentrations significantly increased at 48 h post-inoculation, are displayed within the metabolome of soybean using the SoyCyc tools (Fig. 3*B*). The visualization of pathogen-mediated perturbations of soybean's metabolome using bioinformatics software facilitates not only the global overview and monitoring of the undergoing biochemical processes, but also, applying a reverse genetics approach, the detection of genes that encode the biosynthesis of detected signatory metabolites (Figs. 4 and 5).

### Key Elements of Etiolated Soybean Seedling's Metabolome and Defense Mechanism Against *Rhizoctonia*

**Figure 6:**
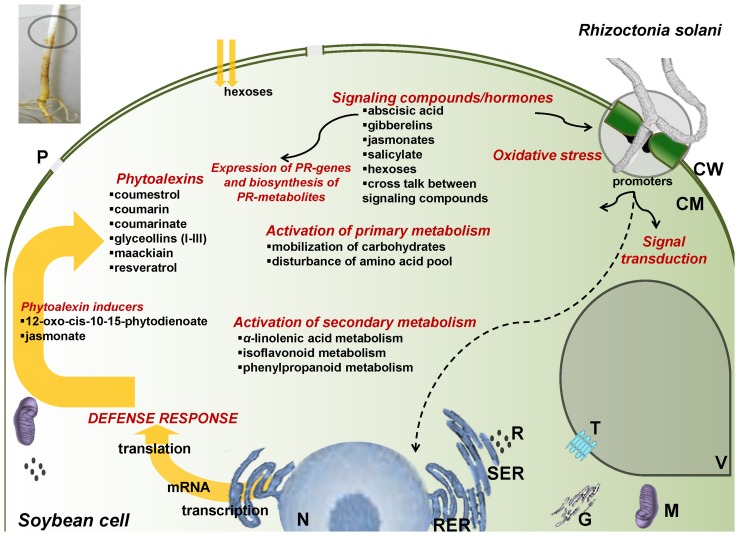
Proposed graphical model for the role of *Rhizoctonia solani* in activation of soybean defense mechanisms. [CM; cell membrane, CW; cell wall, G; Golgi body, M; mitochondrion, N; nucleus, P; plasmalemma, R; ribosomes, RER; rough endoplasmic reticulum, SER; soft endoplasmic reticulum, T; transporters, V; vacuole].

Analysis focused not only on temporal metabolite changes but also on global metabolic network regulation in response to fungal infection (Fig. 6). The most up- and down-regulated soybean pathways are summarized in Table 1.

### *Rhizoctonia* Infection Substantially Alters the Primary Metabolism of Soybean Seedlings

The pathogen attack substantially altered soybean's primary metabolism, as seen by the fluctuation in carbohydrate, amino and carboxylic acids contents (Fig. 4) and instances for up- and down-regulation of amino acid and carbohydrate metabolism at 24 h and 48 h post-inoculation (Table 1, Data Set S1).

*Rhizoctonia* triggered soybean's carbohydrate metabolism causing accumulation of hexose sugars, mainly glucose and fructose, which is in line with previous reports on plant-pathogen pathosystems [22]. This is an indication of altered carbohydrate metabolism following stimulation, with a portion of available hexoses possibly diverted towards the biosynthesis of secondary metabolites [23], and another portion used as an energy source to support the induction of stress-related genes [24].

The protein amino acid pool of seedlings was also substantially affected in two distinct phases, one at 24 h and the second at 48 h post-inoculation. In the first phase, the decrease possibly indicates utilization of amino acids for the biosynthesis of PR proteins and metabolites. A similar trend was reported in the potato-*Rhizoctonia* pathosystem [4]. The situation was reversed in the second phase.

Among non-protein amino acids, the rapid increased levels of *β*-alanine at early stages of infection, also seen in soybean parasitized with aphids [25] and potato sprouts infected with *Rhizoctonia* [4], provide evidence that *β*-alanine may be required in the increased biosynthesis of the coenzyme A (CoA), which plays a central role in various metabolite biosyntheses [26] and contributes to soybean resistance to biotic stress. Early and rapid accumulation of the non-protein amino acid pipecolate, known to act as an osmoprotectant [27] in infected seedlings and a regulator of inducible plant immunity [28] in pathogen-induced systems, is indicative of soybean's response to osmotic stress caused by cell membrane ruptures as early as 24h post-inoculation and priming of the plant to early defense against *Rhizoctonia*.

### *Rhizoctonia* Infection Substantially Alters the Secondary Metabolism of Soybean Seedlings

The secondary metabolism of soybean was also considerably affected by the pathogen's attack as it is indicated by its effect on *α*-linolenate metabolism, isoflavonoid, glucosinolate, and phenylpropanoid biosynthesis, ABC transporters, and protein digestion and absorption (Fig. 2 and Table 1).

#### Regulation of the Isoflavonoid Biosynthetic Pathway

A large number of flavonoids, which are components of the isoflavonoid biosynthetic pathway, were clearly responsive to *Rhizoctonia* infection (Fig. 4, Table 1, Data Set S1) designating it as a primary soybean's defense mechanism against *Rhizoctonia*. Among them, the levels of daidzein, 2′-hydroxydaidzein, 2′-hydroxyformononetin, and maackiain, significantly increased in a concerted fashion following infection.

Daidzein is a central component of the isoflavonoid biosynthetic pathway and it is considered precursor of the soybean's phytoalexins glyceollins and coumestrol and other bioactive flavonoids [29], [30]. Its increased levels led to increase of the phytoalexins coumestrol, and the pterocarpans glyceollins (I-III) (Fig. 4, Data Set S1). The lack of chromatographic separation performing DIMS does not allow separation between glyceollins I-III, therefore from here and throughout the manuscript; the term glyceollins refers to their total relative content. Compared to control, infected seedlings had significantly higher levels of phytoalexins at both time points, with the exception of glyceollins, for which significant difference was observed only at 24 h post-inoculation. In contrast, the less common glyceollin IV was detected at significantly lower levels.

The accumulation of glyceollins and coumestrol in soybean tissues is signal to early defense response to pathogens [31]. Glyceollins are fungitoxic known to restrict pathogen colonization [32], [33]. Also, since they have analogous structures to isoflavonoids, it is likely that their antioxidant activities are similar, thus playing a protective role against reactive oxygen species (ROS) during pathogen attack and conferring partial resistance. Interestingly, glyceollins' levels in *Rhizoctonia-*infected seedlings were not significantly different 48h following infection compared to controls. This, combined with the inability to detect 7-hydroxyglyceollin, a product of glyceollin degradation by pathogens [33], indicates their reduced biosythesis. As with other soybean pathogens [34], challenging soybean with *Rhizoctonia* substantially induces coumestrol biosynthesis whose protective role is attributed to ROS scavenging. Apart from their profound role in soybean physiology, both glyceollins and coumestrol exhibit anti-estrogen and antioxidant activities [35]. Maackiain is another isoflavonoid phytoalexin playing a key role in disease resistance of leguminous crops [36] providing that the attacking pathogen is not able to overcome this defense reaction by degrading it [37]. Here no products of maackiain metabolism were detected.

#### Regulation of the Phenylpropanoid Biosynthetic Pathway

The general disturbance of soybean metabolism is also evident by the fluctuation of metabolites of the phenylpropanoid pathway (Fig. 4, Table 1, and Data Set S1), which plays an important role in plant's physiology, including defense responses, and several of its steps are CyP450-depended [38],[36]. Up-regulation of its branch that leads to the biosynthesis of coumarinate from coumarin was among the most important discoveries. Interestingly, the identified aldehydes of the pathway displayed the same fluctuation pattern, increasing at 24 h post-inoculation, whereas none was detected 48 h later.

Accumulation of several of these metabolites as early as 24 h post-inoculation indicates their involvement in early defense response of soybean. Intriguingly, metabolites of the phenylpropanoid pathway branch that leads to coumarinate were not detected in controls at 48 h after treatments, possibly indicating *de novo* biosynthesis. Coumarins contribute essentially to the persistence of plants being involved in processes such as defense against phytopathogens, response to abiotic stresses, regulation of oxidative stress, and probably hormonal regulation [39]. Caffeate and chlorogenate are phenolics of the pathway with important roles in plant defense mechanism [38], which play a role in the early response to *Rhizoctonia*, plausibly through ROS scavenging [40].

#### Regulation of the α-Linolenic acid Metabolism

The precursor of jasmonate, *α*-linolenate, was detected as another key component of soybean's defense (Fig. 5, Table 1, Data Set S1). The activation of the pathway requires increased levels of *α*-linolenate, which is likely released by complex membrane lipids [41]. Although no discrimination can be achieved between iso-jasmonate and jasmonate (JA), and methyl-jasmonate (MeJA) and isomethyl-jasmonate by DIMS, it is evident that their biosynthesis is an important defense response of soybean. A distinct pattern is observed between the two displayed branches of the pathway. The one that leads to the biosynthesis of JA is activated at 24 h post-inoculation and only JA and MeJA derivatives are detected as signatory metabolites at 48 h. The second that leads to the biosynthesis of traumatin, exhibits a late response to *Rhizoctonia*, since signatory metabolites were detected only at 48 h.

The recognition of elicitor molecules by soybean cell receptors triggers a signal-transduction cascade leading to, among others, the production of signaling compounds such as, JA and its derivatives, 12-oxo-cis-10-15-phytodienoate (OPDA), and traumatin through *α*-linolenate metabolism. Accumulation of jasmonates triggers the biosynthesis of phytoalexins, mediates systemic wound responses, and affects the expression of PR genes synergistically or by antagonizing the action of other plant hormones. JA biosynthesis is regulated by a positive feedback and catalyzed by fatty acid *β*-oxidation enzymes and 4-Cl-like CoA ligases [42]. The biosynthetic intermediate OPDA exhibits stronger phytoalexin-inducing activity compared to JA and MeJA [43]. In this study, elevated levels of OPDA at 24 h post-inoculation indicate its role as an early signal of phytoalexin, JA, and MeJA biosynthesis. Moreover, the accumulation of traumatin in soybeans is possibly associated with lesion healing process of infected seedlings [44].

#### Effect on Various. Metabolites with Important Role in Plant Physiology and Defense

Soybean defense against *Rhizoctonia* is under the regulation of signaling/hormone molecules such as, salicylate (SA), abscisic acid (ABA), JAs, and GAs (Fig. 4, Data Set S1), which are involved in a cross talk [45]. SA is involved in functions related to the systemic acquired resistance (SAR), regulation of defense genes, and programmed cell death. Interestingly, its levels in infected seedlings were lower at 24 h but higher at 48 h post-inoculation, compared to controls. Low levels of SA have been linked to cell death of soybean cell suspensions [45]. The complexity of soybean defense regulation by signaling metabolites/hormones is further confirmed by the increased levels of GAs, which regulate plant growth through the regulation of DELLA family proteins [46], [47], that in turn, affect the balance between SA and JA [48]. Mounting evidence suggests that ABA plays an indecisive role in plant defense responses to pathogens, acting as both a positive and negative regulator of resistance [41], [45]. Here, the increased ABA levels in combination with decreased levels of SA at 48 h, possibly indicates the suppression of SA biosynthesis. Taken together, our results are in line with findings on the involvement of ABA in pathogenesis in the soybean-*Phytophthora sojae* pathosystem [49].

The detection of nucleic acids and components of nucleotide metabolism as signatory metabolites is indicative of the effect of the pathogen on nucleic acid synthesis, energy equilibrium, and biosynthesis of primary and secondary metabolites [50]. As a result of extensive metabolic compartmentation [51], plants require efficient mechanism for resource allocation, which involves passage through various membranes facilitated by ABC transporters [52], [53]. Plants' ABC transporters are not only involved in the transport of plant metabolites and xenobiotics but also contribute to plant–pathogen interactions and the modulation of ion channels [52], [53]. The high number of instances for ABC transporters observed in infected seedlings confirms the intensified operation of the mechanism during soybean-Rhizoctonia interaction.

#### Evidence of reactive oxygen species (ROS) involvement

The biosynthesis of ROS scavengers was also highlighted as an essential component of pathogenesis, confirming ROS involvement during a plant-pathogen interaction, which is not yet fully elucidated [54]. The recognition of the pathogen by plants triggers ROS production, which also plays an important role in virulence and development of pathogens [54]. In addition to the abovementioned metabolites exhibiting antioxidant activities, other metabolites with well-established scavenging properties synthesized during interactions in pathosystems such as, resveratrol [55], ascorbate [56], ornithine [57], and members of the B6 complex [58] were involved in the soybean-*Rhizoctonia* interaction. Furthermore, this is mirrored by increases in glutamate, proline and *γ*-aminobutyric acid (GABA) that can, directly or indirectly, exhibit ROS scavenging activity [59], [60].

## Conclusion

Although the biochemical basis of pathogenesis is extensively studied in plant-pathogen pathosystems, the recent developments in metabolomics now facilitate the comprehensive monitoring of the plant's metabolome and metabolism regulation in response to stimuli, and their study as a whole rather as fragmented pathways. Additionally, although the general knowledge exists for a given plant-pathogen pathosystem, factors related to their genotypic composition could alter the final outcome. Based on its potential, the developed approach could provide new insights and could fill gaps in the knowledge related to the metabolic responses of plants during pathogen invasion.

Additionally, metabolomics data reporting and biological interpretation are facing challenges arising from the inconsistency of chemical names and lack of standardized nomenclature for metabolic pathways across public repositories. The metabolomics strategy reported here enables the robust reporting and biological interpretation of data from untargeted metabolomics experiment by providing standardized overview of the soybean's metabolism regulation during fungal infection, and could be adapted in similar studies. In addition to its significance for plant pathology, results provide information that could be exploited in biomarker-assisted crop breeding, agrochemical, food, and pharmaceutical industries.

## Materials and Methods

### Chemicals and Reagents

Chemicals and reagents of the highest grade commercially available were used. Methoxylamine hydrochloride, *N*-methyl-*N*-(trimethyl-silyl)trifluoroacetamide (MSTFA), and pyridine, for GC/MS sample preparation, and analytical standards, were purchased from Sigma-Aldrich Canada Ltd. (Oakville, ON, Canada). Ethyl acetate, methanol, formic acid, ammonium acetate (Optima grade), and water (HPLC grade), were purchased from Fisher Scientific Company (Ottawa, ON, Canada). For the calibration of analyzers, the ProteoMass ESI Calibration Kit MSCAL5 and MSCAL6 (Sigma-Aldrich), and the DRO/GRO Range Calibration Standard (Restek Corporation, Bellefonte, PA, USA), which is a mixture of 12 alkanes in methylene chloride, were used.

### Biological Material and Inoculation of Etiolated Soybean Seedlings

Seeds of the soybean cultivar AC Proteina (Semican, Plessisville, QC, Canada), which is susceptible to *R. solani*, *were* sterilized by immersion in ethanol (70%, v/v) for 1 min, followed by rinsing three times in sterile distilled water. For pre-germination, seeds were placed on sterilized moistened filter paper (Whatman No1, 11 cm diameter) in Petri plates, and kept at 24°C in the dark. Uniformly pre-germinated seeds were planted in sterile mix of turf:perlite (1∶1, v/v) in plastic trays in the dark in a growth chamber at 22±2°C and relative humidity of 85%. Sterile water was added every second day. Seedlings were etiolated in order to provide longer lengths of hypocotyls for inoculation. They were grown for one week to approximately 15 cm height before they were gently removed and inoculated.

For the production of the inoculum, starter cultures of the highly pathogenic *R. solani* AG4 isolate A76, provided by M. Cubeta (N. Carolina University, USA), were grown on potato dextrose agar (PDA) for three days. Uniform and healthy one-week-old seedlings were transferred to sterile Pyrex glass trays (38×27×6 cm) on filter paper (Whatman No1), arranged horizontally, and their roots were covered with sterile moistened cotton. For the analysis of hyphae, the fungus was grown on cellophane membrane.

Inoculation was performed by sandwiching hypocotyls between two PDA strips (2 cm×8 cm) of 3-day-old *Rhizoctonia* cultures or clean strips (control) (Fig. S3) in sterile environment under photosynthetically inactive black light (365 nm) on designated marked sites of the hypocotyls, 3 cm away from the roots. Trays were wrapped with plastic membrane, and returned to growth chambers for incubation at 24°C, in the dark. The development of necrotic lesions was stereoscopically observed and plants were harvested 24 and 48 h post-inoculation, and prepared for metabolite extraction. These time points were specifically chosen in order to capture the onset of infection and the development of infection cushions. Each treatment (infected or control) was consisted of five biological replications, each obtained by pooling the hypocotyls of four seedlings.

### Sampling and Metabolite Extraction

Samples (100 mg of fresh weight, fw) were harvested from the edges of necrotic lesions at 24 h and 48 h post-inoculation and corresponding portions of control seedlings (Fig. S3). Additionally, mycelia (50 mg, fw) from 3-day-old *R. solani* cultures were harvested. Samples were pulverized to a fine powder in a mortar with a pestle under liquid nitrogen and kept at −80°C in glass autosampler vials (2 ml) until further processing. Metabolite extraction was performed as previously described [4]. Briefly, 1 ml of a mixture of methanol:ethyl acetate (50∶50, v/v) was added to the pulverized samples followed by sonication in an ultrasonic bath Branson 5510 (Branson, Connecticut, USA) for 25 min. Samples were then transferred to an orbital shaker (Infors AG, Bottmingen, Switzerland) for extraction under continuous agitation (150 rpm) for 2 h at 24°C. Extracts were filtered through 0.2 µm filters (Millex-FG, Millipore, MA, USA) to remove debris and the volume of samples was adjusted to 1.0 ml. Consecutively, extracts were divided into two equal portions (0.5 ml) in glass autosampler vials for GC/MS and direct infusion Orbitrap MS analysis (DIMS). The latter were sub-divided into equal portions of 0.25 ml for analysis in positive (ESI^+^) and negative (ESI^−^) electrospray modes, respectively. Finally, extracts were dried using a Labconco CentriVap refrigerated vacuum concentrator (Labconco, Kansas City, MO, USA) equipped with a cold trap.

### Chemical Analyses and Data Pre-processing

#### Direct Infusion Orbitrap MS (DIMS) Analysis

For analyses in ESI^+^, 160 µL of a mixture of methanol:formic acid (0.1% v/v) (50–50, v/v) were added to the dried samples, whereas for analyses in ESI^−^, 160 µL of methanol:ammonium acetate (2.5 mM) were added. Finally, extracts were transferred into microinserters, which were consecutively placed in glass autosampler vials. An LTQ-Orbitrap MS Classic (Thermo Scientific, San Jose, CA, USA) mass spectrometer was employed and experimental events were controlled by the software Xcalibur v.2.2 (Thermo Scientific). The analyzer was equipped with a quadrupole linear ion trap, an Orbitrap electrostatic Fourier transform mass spectrometer (FTMS) with a heated electrospray ionization probe (HESI-II, Thermo Scientific), and an Accela pump (Thermo Scientific). The source voltage was set to 3.2 kV (ESI^+^) and 4.0 kV (ESI^−^) and the capillary voltage to 5.0 V (ESI^+^) and −35 V (ESI^−^). The capillary temperature was set to 275°C. Sheath gas flow was set to 10 (ESI^+^), and 20 (ESI^−^) whereas no auxiliary and sweep gases were used. Samples (10 µL) were injected manually performing direct infusion at a flow rate of 10 µl min^−1^ using a 100 µl syringe (Hamilton, Reno, NV, USA). DIMS analysis was performed at a mass resolution of 60,000 at m/z 400 and spectra were acquired over the range of 50–1,800 Da for 3.5 min. For selected samples MS/MS analyses were performed with the normalized collision energy set at 35 eV, the activation q set to 0.25 and the activation time to 30 ms. Target ions already selected for MS/MS were dynamically excluded for 15 s. AGC target value was set to 5E^5^ for the Orbitrap and to 1E^4^ for the MS/MS analysis in the linear ion trap.

Acquired data (*.raw format) were processed using the software SIEVE v1.3 (Thermo Scientific) installed in a 12-core 64-bit PC workstation (Windows 7 Ultimate SP1, Intel Core i7 CPU, 24 GB RAM). Although the software is designed for the analysis of LC/MS data, with appropriate changes in the settings, we were able to analyse data obtained by DIMS. The acquired mass spectra between 0.6 and 1.2 min were processed (Fig. S5). Obtained frames [rectangular regions of *m/z* vs retention time (RT)] were filtered based on their coefficient of variation distribution (CV) in the reconstructed ion chromatograms (RIC). Frame intensities were normalized to the total ion chromatogram (TIC) and frames with CV>0.5 were excluded from further analysis. The obtained data matrices were exported to Simca P+, after the removal of peaks corresponding to isotopes, for multivariate analyses.

#### Gas Chromatography-Mass Spectrometry (GC/EI/MS) Analysis

Derivatization of samples for GC/MS was performed as previously described [4]. Briefly, methoxymation was performed by adding to the dried samples 80 µL of methoxylamine hydrochloride (20 mg/ml in pyridine) followed by incubation at 30°C for 120 min. In a second step, silylation was performed by adding 80 µL of MSTFA (37°C for 90 min). Derivatized samples were then added to microinserters (150 µL, Fisher Scientific Company), which were placed in autosampler vials (2 ml). For analyses, an Agilent 7890A GC platform (Agilent Technologies Inc. Santa Clara, CA, USA) coupled with a 5975C series mass selective detector (MSD) and a 7693A series autosampler was employed. Electron impact (EI) at 70 eV was used. Full scan mass spectra were acquired at the mass range of 50 to 800 Da at 1 scan s^−1^ rate with a 10.0-min solvent delay. The temperature for the ion source was set to 150°C, for the transfer line to 230°C, and for the injector to 230°C. Samples (1 µL) were injected using a split ratio of 10:1 into a HP-5MS ultra inert (UI) capillary column (30 m×250 µm I.D, 0.25 µm film thickness; Agilent Technologies Inc.). Helium was used as the carrier gas at a constant flow rate of 1 ml min^−1^. The temperature of the oven was initially 70°C stable for 5 min, followed by a 5°C min^−1^ increase to 310°C and finally stable for 1 min.

For data pre-processing (peak deconvolution, metabolite identification, and peak integration) the Agilent MSD Chemstation E.02.00.493 was used. Automatic integration was performed after optimization of the ChemStation Integrator parameters based on the total ion chromatogram (TIC). Processed chromatograms were exported to Microsoft excel for the creation of data matrices. Following retention time (RT) alignment, chromatograms were normalized to the TIC and the constructed matrix was exported to Simca P+ v.12.0.1 for the discovery of signatory metabolites and trends. To ensure the quality and validity of analyses, standard operating procedures (SOP) and quality control (QC) measures were applied throughout analyses.

#### Metabolite Identification and Construction of Standardized Metabolite Library for Soybean

In order to develop a high-throughput metabolomics protocol, identification was performed following a biologically-driven approach. Central to this approach is the species-specific in-house built library for soybean composed of 1339 metabolites, which includes information on metabolite monoisotopic mass, molecular formula, biosynthetic pathways, and chemical classification. For the construction of the library, information was retrieved from the publicly available databases of SoyCyc (http://www.soybase.org:8082/SOY/class-instances?object=Compounds), the Kyoto Encyclopedia of Genes and Genomes (KEGG, http://www.genome.jp/kegg/), PubChem (http://pubchem.ncbi.nlm.nih.gov/), the European Bioinformatics Institute (EMBL-EBI, http://www.ebi.ac.uk/), KNApSAcK (http://kanaya.naist.jp/KNApSAcK/), and the literature. For standardization purposes, the KEGG coding system for pathways was adapted, which enables the comprehensive grouping of pathways and is compatible with bioinformatics tools such as the pathway tools of BioCyc (http://bioinformatics.ai.sri.com/ptools/). For the chemical classification of metabolites, information was retrieved from the database PubChem (http://pubchem.ncbi.nlm.nih.gov/).

For Orbitrap MS analyses, using the software SIEVE, searches for the constructed frames were performed against the constructed library with a mass error (*Δppm*) <2 ppm. Putative identification of metabolites was based on mass accuracy and, where available, MS/MS fragmentation and isotope patterns, following heuristic rules [61]. MS/MS spectra deconvolution was performed using the software MetWorks 1.3 SP3 and Mass Frontier 7.0 SR1 (Thermo Scientific).

For GC/MS, the guidelines of the metabolomics standards initiative (MSI) [8] were followed. Mass spectra searches were performed against the library of the National Institute of Standards and Technology (Gaithersburg, MD, USA) NIST 08 and for selected signatory metabolites, absolute identification was based on fragmentation patterns and retention times (RT) analyzing authentic chemical standards on the same GC/MS system with the same analytical method [8]. In cases in which a very good fit (e.g,>90%) could be achieved, tentative identification was performed. Metabolites of non-biological origin were excluded from further analysis.

#### Statistical Analyses

For Orbitrap MS data, one-way ANOVA was executed using the integrated statistical function of SIEVE v.1.3 performing the Student's t-test (*P*<0.05). GC/MS data matrices were subjected to multivariate analyses using the software SIMCA-P+ v.12.0 (Umetrics, MKS Instruments Inc. Andover, MA, USA) as previously described (4) and one-way ANOVA using the software JMP 11.0 (SAS Institute Inc, NC, USA) performing the Student's t-test (*P*<0.05). The discovery of signatory metabolites performing multivariate analysis was based on partial least square-discriminant analysis (PLS-DA) regression coefficients (*P*<0.05). Standard errors were calculated using Jack-knifing, which is based on the variability in the model parameters encountered in the different cross-validation cycles with 95% confidence interval [62]. The performance of the models was assessed by the cumulative fraction of the total variation of the *X*'s that could be predicted by the extracted components [*Q^2^_(cum)_*] and the fraction of the sum of squares of all *X*'s (*R^2^X*) and *Y*'s (*R^2^Y*) explained by the current component.

Analysis of metabolic networks The global soybean metabolome was visualized using the software Cytoscape v.2.8.2 (http://www.cytoscape.org/) [63] and the SoyCyc database as previously described [4]. By integrating information from analyses, enzymes catalyzing the biosynthesis of metabolites that are signatory of the soybean's response to *Rhizoctonia* infection and corresponding encoding genes were inferred and highlighted. Additionally, the soybean's metabolome fluctuation in response to pathogen attack was visualized using the cellular overview tool of SoyCyc (http://www.soybase.org:8082/overviewsWeb/celOv.shtml) and information from the in-house built library. The Data Set S5 was used.

#### Quality Control of Metabolomics Analyses

For each treatment, a quality control (QC) sample was obtained by pooling 25 µL from each of the five biological replicates (total 125 µL), and analyzed. In addition to biological replications and QC samples, technical one replication for randomly selected samples was performed per treatment. Following each sample's analysis, a blank (solvent system only) was injected to ensure that no carry over is observed between samples. Additionally, blank samples were used for the detection of possible contamination during the different experimental steps, such as material and reagent impurities and column stationary phase bleeding (for GC/MS). For this purpose, 1.5 mL of the extraction solvent mixture was processed alongside the experimental samples and was subjected to identical handling. Results were taken into account for the detection of metabolites not related to the biological material that was analyzed, which were subsequently excluded from analyses. Additionally, the metabolic composition of fungal samples was taken into consideration, in order to remove fungal-derived metabolites that could influence the biological interpretation of results. In order to optimize the performance of analyzers, calibration was performed at the beginning of analyses following the recommended by the manufacturers' procedures and using calibration solutions.

For GC/MS, tuning of the MS detector was performed automatically using the AutoTune function of the Agilent MSD Chemstation. The DRO/GRO Range Calibration Standard was injected every six samples in order to monitor the performance of the system and additionally, samples were spiked by adding 20 µL of ribitol solution (0.2 mg/mL) to methanol-water (50–50, v/v), which served as the internal standard.

For Orbitrap MS, the ProteoMass ESI Calibration Kit MSCAL5 (Sigma-Aldrich), which covers the range between 138 and 1822 Da, was used for ESI^+^ analyses. For ESI^−^, calibration was performed using the ProteoMass ESI Calibration Kit MSCAL6 (Sigma-Aldrich), which covers the range between 265 to 1880 Da. Internal standards were not used but instead, mass errors were estimated based on accurate masses of common identified metabolites such as amino (ESI^+^) and lipid (ESI^−^) acids.

## Supporting Information

Figure S1 **Partial least squares-discriminant analyses (PLS-DA) PC1/PC2 score plots of direct infusion Orbitrap MS and GC/MS metabolite profiles of control (▪) and** ***Rhizoctonia solani*** **-infected (•) soybean seedlings, at 24 h and 48 h post-inoculation.** The ellipse represents the Hotelling T^2^ with 95% confidence interval. Five (5) biological replications were used per treatment and one quality control sample (QC) [Q^2^_(cum)_; cumulative fraction of the total variation of the X's that can be predicted by the extracted components, *R^2^X* and *R^2^Y*; the fraction of the sum of squares of all *X*'s and *Y*'s explained by the current component, respectively, *PCs*; principal components].
(TIF)

Figure S2 **Pipeline for the dissection of plant-pathogen pathosystems performing high-throughput metabolomics using as model the pathosystem soybean-** ***Rhizoctonia solani.***
(TIF)

Figure S3 **Experimental set up for the infection of soybean seedlings with** ***Rhizoctonia solani*** **AG4.** For the inoculation, three-day old cultures of *Rhizoctonia* grown on PDA were used (*A*). The basal portion of seedlings was sandwiched between two strips of PDA (control) (*B*) or *Rhizoctonia* culture (*C*) and the roots were sandwiched between two layers of sterile moistened cotton. Seedlings were kept in sterilized Pyrex trays on filter paper and sealed with cellophane membrane. Samples were taken at 24 h (*D*) and 48 h (*E*) post-inoculation from the edges of the necrotic lesions and corresponding segments of control seedlings.
(TIF)

Figure S4 **Chemical structures of representative soybean's signatory metabolites in response to** ***Rhizoctonia solani*** **at 24 h and/or 48 h post-inoculation.**
(TIF)

Figure S5 **Representative total ion chromatograms (TIC) of control and** ***Rhizoctonia solani*** **-infected soybean seedlings at 24 h and 48 h post-inoculation performing direct infusion Orbitrap MS analysis.** Mass spectra that correspond to the area between the dashed lines were used in metabolomics analyses.
(TIF)

Table S1 Number of frames of direct infusion Orbitrap MS using the software SIEVE v.1.3.
(DOC)

Data Set S1 **Identified metabolites signatory of the soybean defense against** ***Rhizoctonia solani*** **based on LTQ Orbitrap and/or GC/MS analyses.** With red color metabolites whose relative concentration is increased in infected seedlings and with green color, those whose relative concentration is decreased compared to controls.
(XLS)

Data Set S2 **GC/MS spectra library.** GC/MS mass spectra library (*.MSL) of identified metabolites of biological origin. The library can be visualized using the freely available software AMDIS (http://chemdata.nist.gov/mass-spc/amdis/downloads/). Information on the retention times and analytical conditions are provided. The library should be downloaded and then viewed by selecting “Build one library” under the “Library” tab of the software.
(MSL)

Data Set S3 **DIMS Orbitrap MS/MS spectra.** Recorded MS/MS spectra of representative chromatogram from Soybean seedlings infected by *Rhizoctonia solani* at 48 h post-infection in the positive electrospray mode between 0.6 and 1.2 min.
(TXT)

Data Set S4 **DIMS Orbitrap MS/MS spectra.** Recorded MS/MS spectra of representative chromatogram from Soybean seedlings infected by *Rhizoctonia solani* at 48 h post-infection in the negative electrospray mode between 0.6 and 1.2 min.
(TXT)

Data Set S5 **Data set for the visualization of identified signatory metabolites within the global metabolome of soybean using the cellular overview tool of SoyCyc.** Kegg identifiers for metabolites (first column), and relative composition of metabolites in control (second column) and infected by *Rhizoctonia solani* soybean seedlings (third column), at 48 h post-inoculation are displayed.
(TXT)
