# Supplementary figures and images for: A Metabolic Profiling Strategy for the Dissection of Plant Defense against Fungal Pathogens
Source: PLoS One. 2014 Nov 4;9(11):e111930. doi: 10.1371/journal.pone.0111930 (PMC4219818; doi:10.1371/journal.pone.0111930)

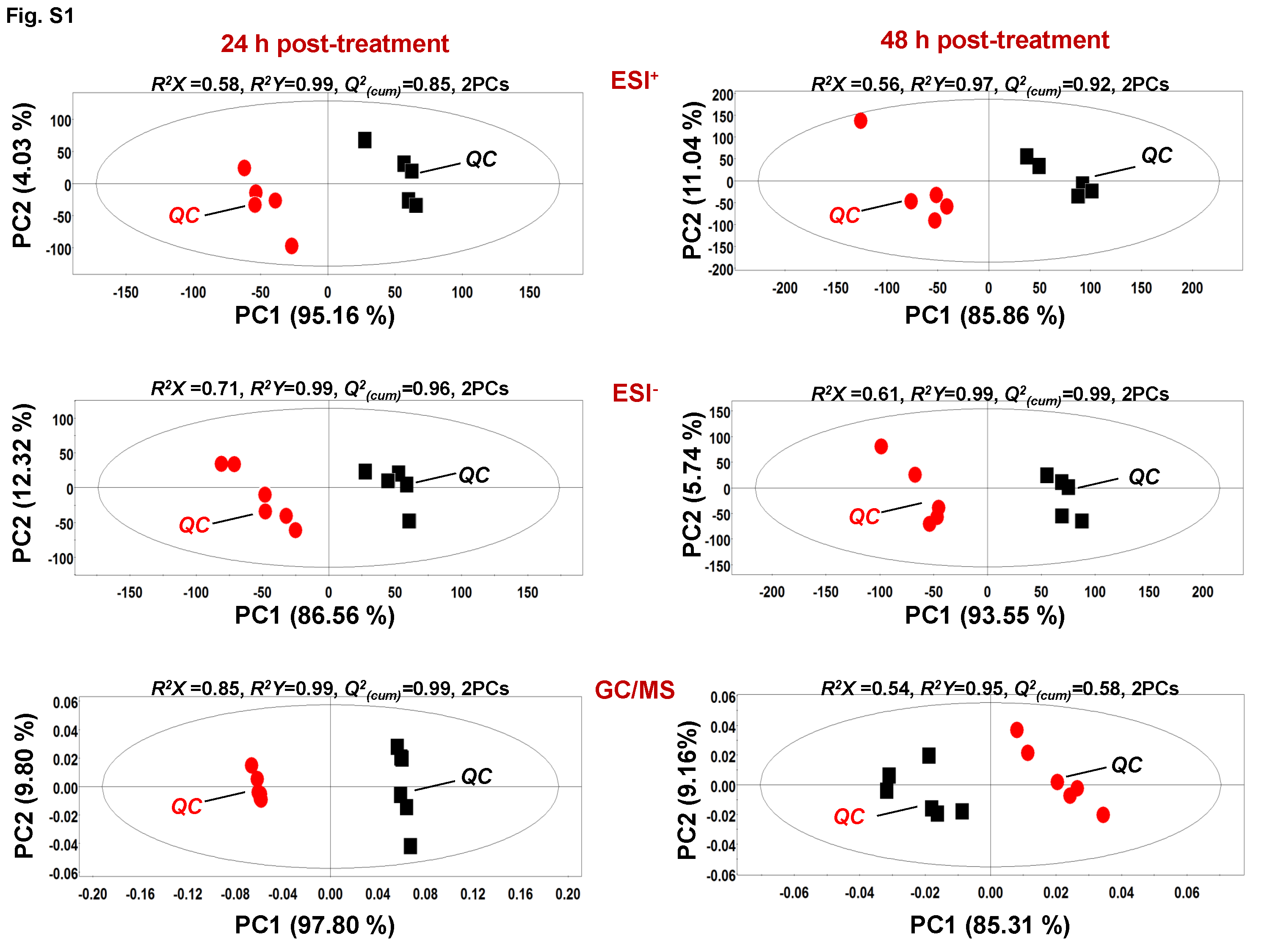

Supplement: Figure S1 — Partial least squares-discriminant analyses (PLS-DA) PC1/PC2 score plots of direct infusion Orbitrap MS and GC/MS metabolite profiles of control (▪) and Rhizoctonia solani -infected (•) soybean seedlings, at 24 h and 48 h post-inoculation. The ellipse represents the Hotelling T2 with 95% confidence interval. Five (5) biological replications were used per treatment and one quality control sample (QC) [Q2 (cum); cumulative fraction of the total variation of the X's that can be predicted by the extracted components, R2X and R2Y; the fraction of the sum of squares of all X's and Y's explained by the current component, respectively, PCs; principal components]. (TIF) [file pone.0111930.s001.tif]

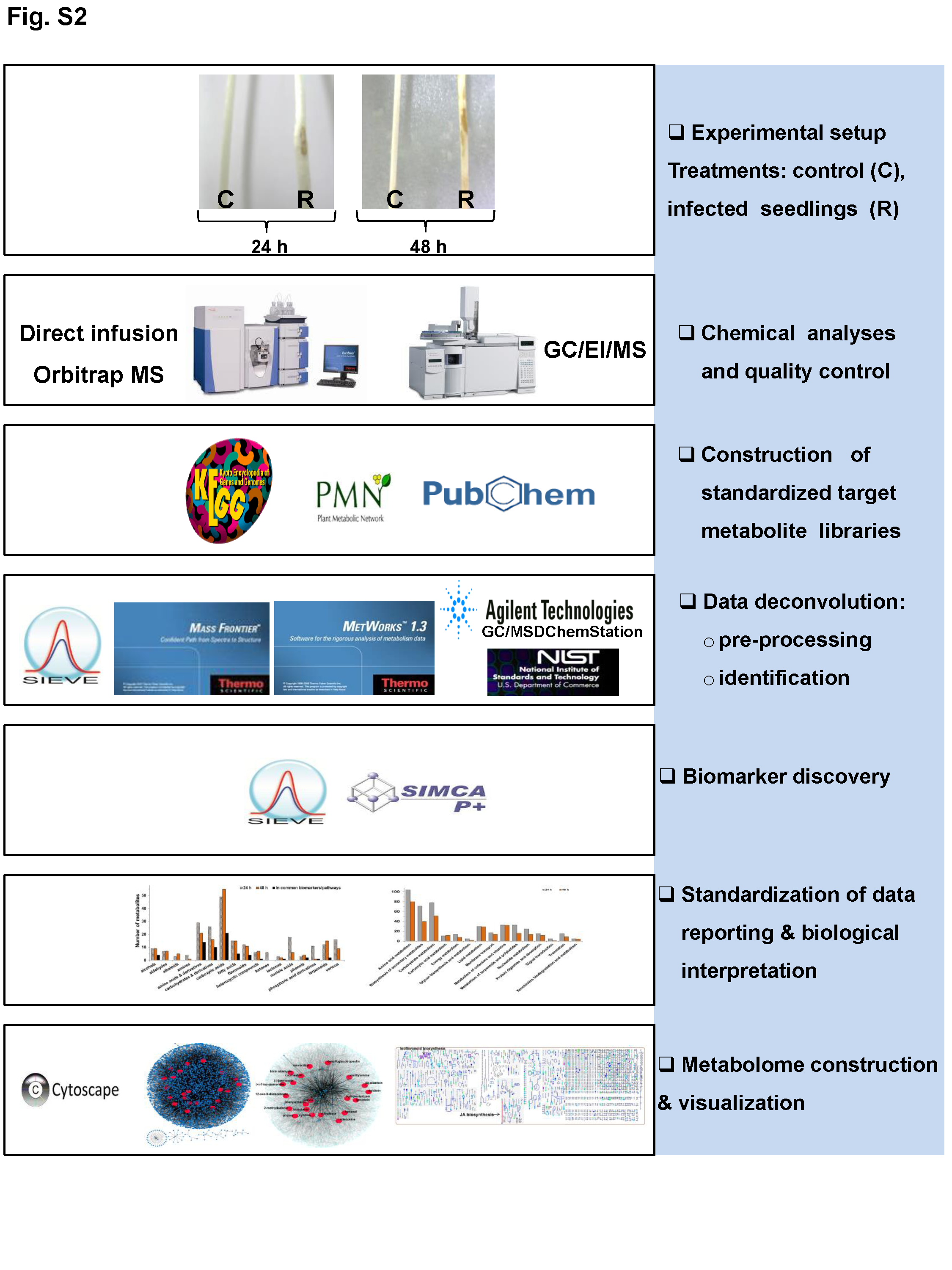

Supplement: Figure S2 — Pipeline for the dissection of plant-pathogen pathosystems performing high-throughput metabolomics using as model the pathosystem soybean- Rhizoctonia solani. (TIF) [file pone.0111930.s002.tif]

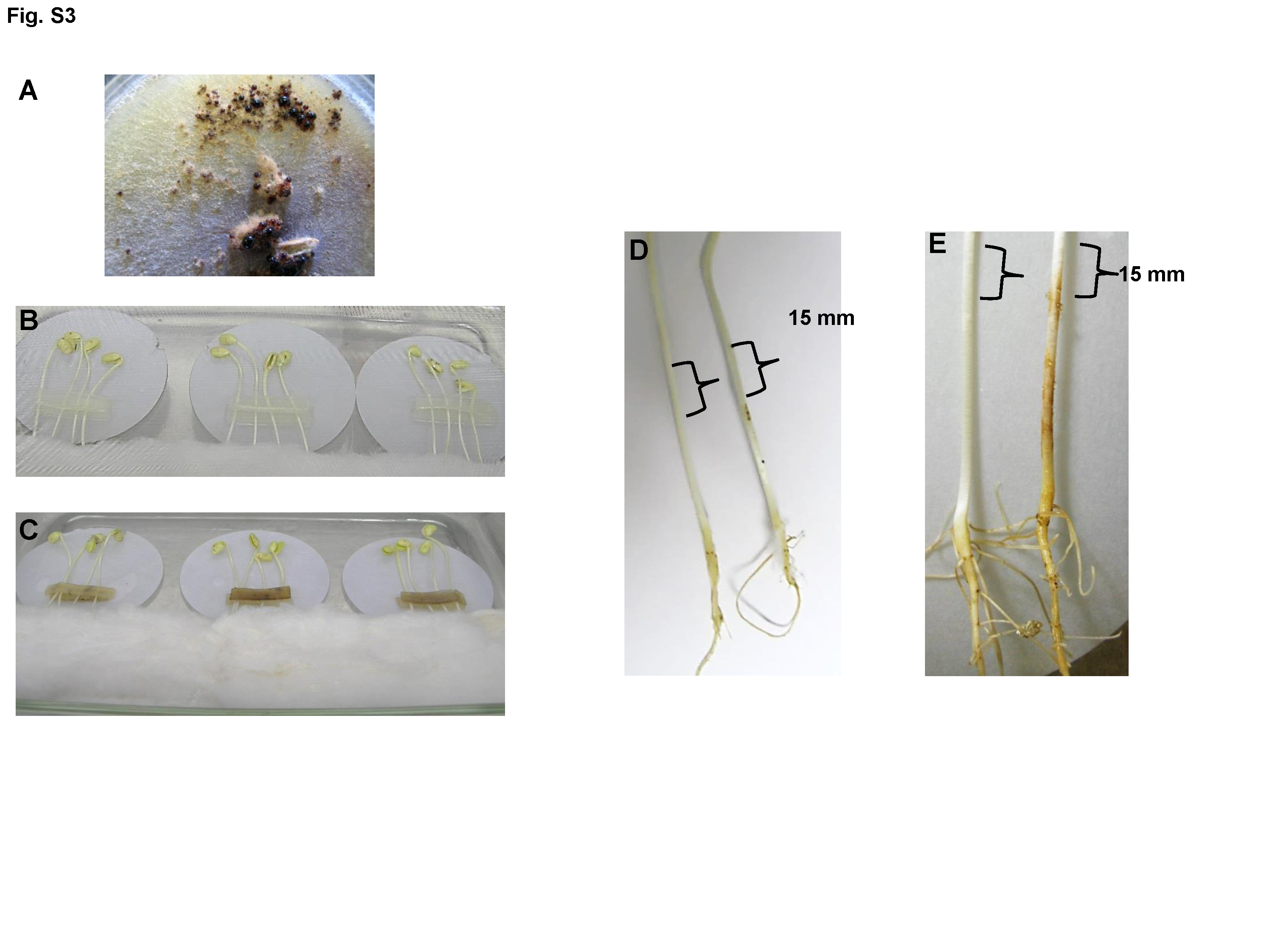

Supplement: Figure S3 — Experimental set up for the infection of soybean seedlings with Rhizoctonia solani AG4. For the inoculation, three-day old cultures of Rhizoctonia grown on PDA were used (A). The basal portion of seedlings was sandwiched between two strips of PDA (control) (B) or Rhizoctonia culture (C) and the roots were sandwiched between two layers of sterile moistened cotton. Seedlings were kept in sterilized Pyrex trays on filter paper and sealed with cellophane membrane. Samples were taken at 24 h (D) and 48 h (E) post-inoculation from the edges of the necrotic lesions and corresponding segments of control seedlings. (TIF) [file pone.0111930.s003.tif]

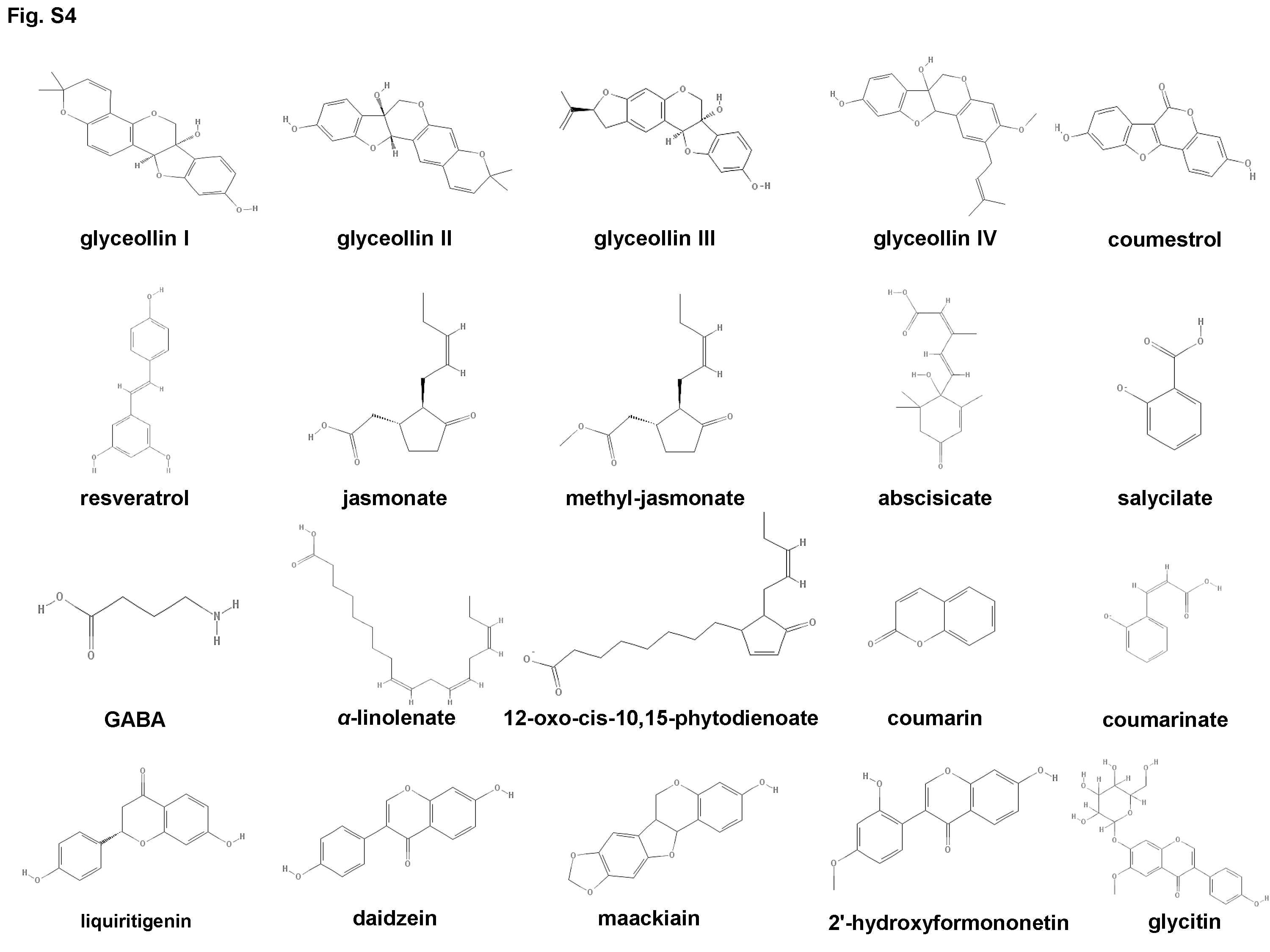

Supplement: Figure S4 — Chemical structures of representative soybean's signatory metabolites in response to Rhizoctonia solani at 24 h and/or 48 h post-inoculation. (TIF) [file pone.0111930.s004.tif]

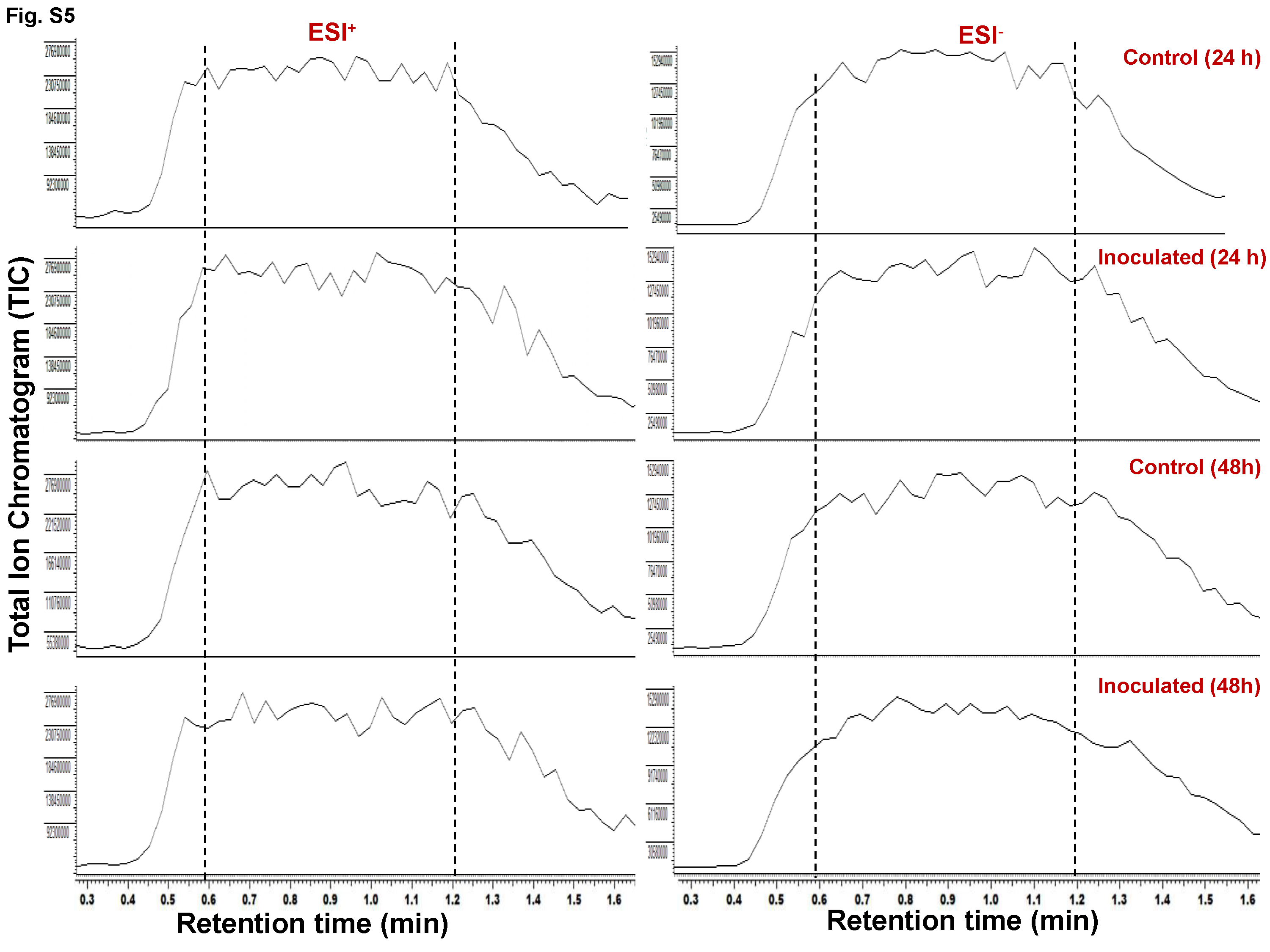

Supplement: Figure S5 — Representative total ion chromatograms (TIC) of control and Rhizoctonia solani -infected soybean seedlings at 24 h and 48 h post-inoculation performing direct infusion Orbitrap MS analysis. Mass spectra that correspond to the area between the dashed lines were used in metabolomics analyses. (TIF) [file pone.0111930.s005.tif]
