# Supplementary material for: A Metabolic Profiling Strategy for the Dissection of Plant Defense against Fungal Pathogens
Source: PLoS One. 2014 Nov 4;9(11):e111930. doi: 10.1371/journal.pone.0111930 (PMC4219818; doi:10.1371/journal.pone.0111930)
Supplement: Table S1 — Number of frames of direct infusion Orbitrap MS using the software SIEVE v.1.3. (DOC) [file pone.0111930.s006.doc]

**Table S1. Number of frames of direct infusion Orbitrap MS data acquired in positive (ESI^+^) and negative (ESI^-^) electrospray modes using the software SIEVE v1.3, 24 and 48 h following infection of soybean seedlings with *Rhizoctonia solani*.**

| **ESI/Treatment** | **Intensity threshold*^a^*** | **Original number of frames*^b^*** | **Number of frames after filtering*^c^*** | **Number of frames *P*<0.05*^d^*** |
| --- | --- | --- | --- | --- |
| ESI^+^/24h post-infection | 23000 | 52141 | 15747 | 2417 |
| ESI^+^/48h post-infection | 23000 | 63187 | 3044 | 1228 |
| ESI^-^/24h post-infection | 15000 | 34336 | 9584 | 1619 |
| ESI^-^/48h post-infection | 15000 | 39354 | 4057 | 1322 |

*^a^*absolute value of intensities

*^b^*rectangular regions of *m/z* vs retention time (RT)

*^c^*filtering; removal of isotopes and frames with coefficient of variation distribution (CV) <0.5

*^d^P*-value performing the Student’s t-test
